# Coding-complete genome sequences of hedgehog coronavirus isolated from *Erinaceus europaeus* in France

**DOI:** 10.1128/mra.00157-25

**Published:** 2025-06-09

**Authors:** Kevyn Beissat, Evelyne Picard-Meyer, Pascal Arné, Flavie Chanteclair, Cécile Le Barzic, Lorette Hivert, Guillaume Le Loc'h, Marie-Pierre Puech, Fanny Bastien, Jean-Luc Schereffer, Marine Wasniewski

**Affiliations:** 1 Nancy Laboratory for Rabies and Wildlife, ANSES, Malzéville, France; 2 USC-1233 Rongeurs Sauvages Risques Sanitaires et Gestion des Populations (RS2GP), VetAgroSup, Institut National de Recherche pour l’Agriculture, l’Alimentation et l’Environnement (INRAE), Lyon University, Marcy-L'Etoile, France; 3 Centre Hospitalier Universitaire Vétérinaire de la Faune Sauvage (Chuv-FS), Ecole Nationale Vétérinaire d’Alfort, Maisons-Alfort, France; 4 UMR 1161 Virologie, INRAE-ENVA-ANSES, Ecole Nationale Vétérinaire d’Alfort, Maisons-Alfort, Paris, France; 5 Wildlife Care Centre, Ecole Nationale Vétérinaire de Toulouse (ENVT), Université de Toulouse, Toulouse, France; 6 IHAP, ENVT, INRAE, Université de Toulouse, Toulouse, France; 7 Hôpital Faune Sauvage - Goupil connexion, Ganges, France; Queens College Department of Biology, Queens, New York, USA

**Keywords:** hedgehog, coronavirus, complete genome, *Erinaceus europaeus*

## Abstract

The coding-complete genome sequences of hedgehog coronavirus were retrieved from rectal swabs collected in 2022 and 2023 in France. Information on this virus is crucial to understand the pathogenicity and ecology of this *Betacoronavirus*.

## ANNOUNCEMENT

The hedgehog coronavirus (*Coronaviridae* family, *Betacoronavirus* genus, *Merbecovirus* subgenus), otherwise known as the *Erinaceus* coronavirus (EriCoV), was first detected in fecal samples of European Hedgehog (*Erinaceus europaeus*) in 2012 in Germany (1). EriCoV is capable of infecting hedgehogs exclusively and seems to be asymptomatic (2). This virus groups phylogenetically with bat coronaviruses and MERS-CoV (Middle-East Respiratory Syndrome Coronavirus), which is also known to infect humans (1, 3). To date, two hedgehog coronaviruses have been identified. The first EriCoV has been detected in *E. europaeus* in several European countries (1–7). The second EriCoV, a slightly different strain (HKU31), has been detected in the Amur Hedgehog (*Erinaceus amurensis*) in China (8, 9). Complete genomes of EriCoV have already been obtained in Europe (Germany, the United Kingdom, Poland, Italy, and Russia).

Two coding-complete genomes were retrieved from French European hedgehogs, collected in 2023 in Toulouse, Haute Garonne, and 2022 in Maisons-Alfort, Val-de-Marne.

These sequences were isolated from rectal swabs (Σ-VIROCULT) collected between 2020 and 2023 from three wildlife care centers located in Toulouse, Maisons-Alfort, and Ganges in France. Sample collection was authorized by the ANSES ethics committee (ComEth). RNA was extracted using the NucleoSpin RNA, Mini Kit (Macherey-Nagel). Following primers (PanCov Pol 15197-Forward: GGTTGGGACTATCCTAAGTGTGA and PanCov Pol 15635-Reverse: CCATCATCAGATAGAATCATCAT) (10) were used to perform RT-PCR and nested PCR targeting the RNA-dependent RNA polymerase (RdRp) gene (11) of alphacoronaviruses and betacoronaviruses.

Next-generation sequencing was performed on total RNA by Helixio, France, using a 2 × 150 bp sequencing library prepared on Illumina with the NEBNext Ultra II Directional RNA Library Prep Kit (New England Biolabs). A total of 15.9 and 15 million reads were generated for the two samples. MultiQC v.1.27 (12) was used to check the quality of all total reads. Trimmomatic v.0.39 (13) was utilized to remove adapters and perform a quality check of raw reads. Raw reads were cut from 5′ and 3′ extremities if quality was under 20, and reads were deleted if their length was under 90 bp.

*De novo* assembly was performed using RNA SPAdes v.3.14.0 (14), and quality was checked using QUAST v.5.0.2 (15). Unless otherwise indicated, default parameters were utilized. A total of 55,017 (22-3151 sample) and 74,536 (22-7137 sample) sequences were aligned with the reference sequence (NC_039207). Only four and one sequences, respectively, for the 22-3151 and 22-7137 samples uniquely mapped to the reference sequence.

**Fig 1:**
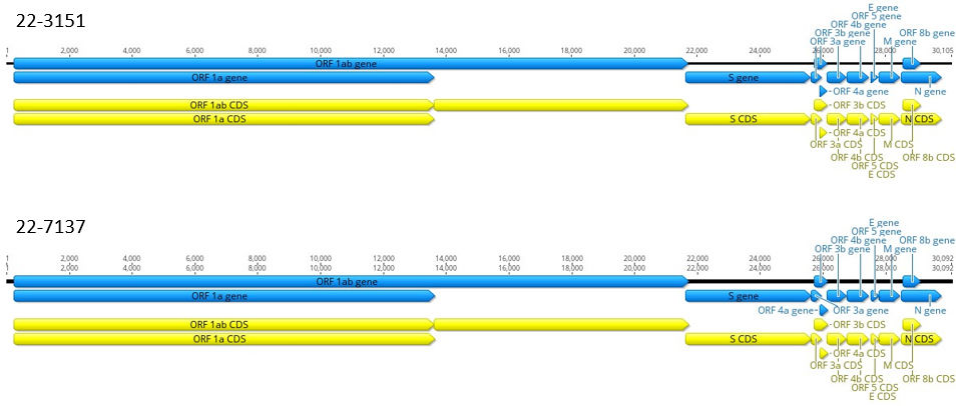
Genome organization of the EriCoV full genome sequences (samples 22-3151 and 22-7137) isolated in *Erinaceus europaeus* in France.

The coding regions were visualized and annotated using Geneious Prime v.2022.0.2 (16) by referring to the reference sequence. The genomic organization of both EriCoV sequences was found to be similar to EriCoV isolated in Europe, with a complete coding-genome length of 30,105 nucleotides for 22-3151 (37.6% GC content) and 30,092 nucleotides for 22-7137 (37.5% GC content) (Fig. 1). Using BLAST v.2.16.0 (17), 22-3151 and 22-7137 were, respectively, 93.64% and 93.68%, similar to the reference sequence.

## Data Availability

EriCoV sequences have been deposited in GenBank (PQ869634 and PQ869635). The raw sequence reads are accessible in the Sequence Read Archive (SRA) (SRR31931794 and SRR31931795).
